# Intranasally Administered L-Myc-Immortalized Human Neural Stem Cells Migrate to Primary and Distal Sites of Damage after Cortical Impact and Enhance Spatial Learning

**DOI:** 10.1155/2021/5549381

**Published:** 2021-05-22

**Authors:** Margarita Gutova, Jeffrey P. Cheng, Vikram Adhikarla, Lusine Tsaturyan, Michael E. Barish, Russell C. Rockne, Eleni H. Moschonas, Corina O. Bondi, Anthony E. Kline

**Affiliations:** ^1^Department of Developmental & Stem Cell Biology, Beckman Research Institute, City of Hope, Duarte, CA, USA; ^2^Physical Medicine & Rehabilitation, University of Pittsburgh, Pittsburgh, PA, USA; ^3^Safar Center for Resuscitation Research, University of Pittsburgh, Pittsburgh, PA, USA; ^4^Department of Computational and Quantitative Medicine, Division of Mathematical Oncology, Beckman Research Institute, City of Hope, Duarte, CA, USA; ^5^Center for Neuroscience, University of Pittsburgh, Pittsburgh, PA, USA; ^6^Neurobiology, University of Pittsburgh, Pittsburgh, PA, USA; ^7^Center for the Neural Basis of Cognition, University of Pittsburgh, Pittsburgh, PA, USA; ^8^Critical Care Medicine, University of Pittsburgh, Pittsburgh, PA, USA; ^9^Psychology, University of Pittsburgh, Pittsburgh, PA, USA

## Abstract

As the success of stem cell-based therapies is contingent on efficient cell delivery to damaged areas, neural stem cells (NSCs) have promising therapeutic potential because they inherently migrate to sites of central nervous system (CNS) damage. To explore the possibility of NSC-based therapy after traumatic brain injury (TBI), isoflurane-anesthetized adult male rats received a controlled cortical impact (CCI) of moderate severity (2.8 mm deformation at 4 m/s) or sham injury (i.e., no cortical impact). Beginning 1-week post-injury, the rats were immunosuppressed and 1 × 10^6^ human NSCs (LM-NS008.GFP.fLuc) or vehicle (VEH) (2% human serum albumen) were administered intranasally (IN) on post-operative days 7, 9, 11, 13, 15, and 17. To evaluate the spatial distributions of the LM-NSC008 cells, half of the rats were euthanized on day 25, one day after completion of the cognitive task, and the other half were euthanized on day 46. 1 mm thick brain sections were optically cleared (CLARITY), and volumes were imaged by confocal microscopy. In addition, LM-NSC008 cell migration to the TBI site by immunohistochemistry for human-specific Nestin was observed at day 39. Acquisition of spatial learning was assessed in a well-established Morris water maze task on six successive days beginning on post-injury day 18. IN administration of LM-NSC008 cells after TBI (TBI + NSC) significantly facilitated spatial learning relative to TBI + VEH rats (*p* < 0.05) and had no effect on sham + NSC rats. Overall, these data indicate that IN-administered LM-NSC008 cells migrate to sites of TBI damage and that their presence correlates with cognitive improvement. Future studies will expand on these preliminary findings by evaluating other LM-NSC008 cell dosing paradigms and evaluating mechanisms by which LM-NSC008 cells contribute to cognitive recovery.

## 1. Introduction

Traumatic brain injury (TBI) often results in long-term neurological disabilities [1–3] and affects ten million individuals worldwide each year [4]. In the United States, the incidence is 2.8 million per year [5]. Although motor function is negatively impacted, preclinical and clinical evidence indicates that cognitive impairments are more pronounced and prolonged [6–12] and adversely affect quality of life [13, 14]. The economic cost of acute medical care and subsequent rehabilitation, along with loss of productivity due to the inability to return to full-time work, is billions of dollars each year [3]. TBI is a significant health care concern, and thus, numerous pharmacological interventions have been evaluated [15–18]. Although various approaches have shown benefits in the laboratory, successful clinical translation has been essentially nonexistent [19, 20]. This dismal situation has motivated the development of other alternative therapeutic strategies.

Neural stem cells (NSCs) are attractive candidates for restoration of brain function after TBI as they inherently migrate to damaged sites where they contribute neurotrophic factors to suppress inflammation, protect against further neuronal loss, promote recovery of existing damaged neurons, and possibly replace lost neurons and other cells [21–24]. A major challenge to successful NSC-based therapy is ensuring that sufficient numbers of cells reach damaged and nonfunctioning regions. To address this issue, we and others have explored delivery of NSCs to the central nervous system (CNS) by intravenous (IV) and intracranial (IC) injection and by intranasal (IN) inhalation [25–27]. Although IV-injected NSCs localize to damaged tissue, they show limited accumulation (i.e., less than 1% of injected NSCs) in the brain [27, 28]. IV administration of NSCs can also trigger adverse immune responses and other systemic complications. IC administration of NSCs avoids potential systemic reactions [27], but it is invasive, inefficient, costly, and potentially damaging to normal brain and places patients at greater risk. The inherent limitations of IV and IC administration support the evaluation of IN delivery, which is less invasive and can be performed repeatedly [23].

The aim of the current study was to determine whether IN-administered L-MYC-immortalized human NSCs (LM-NSC008 cells) accumulate in damaged brain tissue and facilitate cognitive recovery in a well-established rat model of TBI [6, 29–31]. To accomplish this aim, six doses of LM-NSC008 cells were administered once every other day beginning one week after cortical impact injury or sham surgery to adult rats, and patterns of early and late NSC migration were assessed as was the acquisition of spatial learning in a validated Morris water maze task. The findings indicate that IN-delivered NSCs migrate to primary and distant TBI sites and facilitate acquisition of spatial learning. As NSC-based therapy can be delivered IN, it is a potentially feasible alternative to pharmacotherapies for cognitive recovery after TBI. If this therapeutic approach can be successfully translated to the clinic, it would allow for extended, cost-effective, and relatively noninvasive treatments for TBI patients.

## 2. Materials and Methods

### 2.1. Subjects

Forty-eight adult male Sprague-Dawley rats (Envigo, Indianapolis, IN) weighing 300–350 g on the day of surgery were housed in pairs in Plexiglas cages with *ad libitum* food and water and maintained in a temperature- (21 ± 1°C) and light- (on 0700 to 1900) controlled environment. After a week of acclimatization, the rats were randomly assigned to one of four experimental groups: TBI + NSC (*n* = 17), TBI + VEH (human serum albumin; *n* = 16), sham + NSC (*n* = 6), and sham + VEH (*n* = 9). An additional 10 adult female rats weighing 250–275 g on the day of surgery were included for IHC staining using anti-human Nestin-specific antibodies to visualize LM-NSC008 cells at the TBI site on day 39—TBI + NSC (*n* = 3), TBI + VEH (*n* = 3), sham + NSC (*n* = 2), and sham + VEH (*n* = 2). Procedures for TBI and sham surgeries, IN administrations, and cognitive assessments were approved by the University of Pittsburgh's Institutional Animal Care and Use Committee and the Institutional Biosafety Committee.

### 2.2. Surgery

A controlled cortical impact (CCI) injury was produced as previously described [6, 8, 9, 29, 31–33]. Briefly, rats were fully anesthetized with inspired isoflurane (4% induction and 2% maintenance with a 2 : 1 ratio of N_2_O : O_2_), intubated, fixed in a stereotaxic frame, and ventilated mechanically. Adhering to aseptic technique, a midline scalp incision was made to expose the skull and a craniectomy was made in the right hemisphere between bregma and lambda and the sagittal and coronal sutures with a high-speed dental drill. The bone flap was removed, and the surgical opening was enlarged further to ensure clearance of the impact tip (6 mm, flat), which was centered and lowered through the craniectomy until it touched the dura mater. The rod was then retracted, and the impact tip was advanced 2.8 mm to produce a moderate brain injury (2.8 mm tissue deformation at 4 m/s). Following impact, the rats were sutured, extubated, assessed for acute neurological function (flexion and righting reflexes), and allowed to recover spontaneous ambulation prior to being returned to their home cage. The core temperature was maintained at 37°C ± 0.5°C with a heating blanket. Sham controls underwent all procedures except for the CCI.

### 2.3. Acute Neurological Evaluation

Hind limb reflexive ability was assessed after the cessation of anesthesia and removal from the stereotaxic apparatus by gently squeezing the rats' hind paws every 5 s and recording the time to elicit a withdrawal response. The return of the righting reflex was determined by the time required to turn from the supine to prone position three consecutive times. These neurological indices are sensitive determinants of injury severity [6, 8, 9, 33–35].

### 2.4. Administration of LM-NSC008 Cells or VEH

Beginning 1 week after TBI or sham surgery, the rats were IN administered1 × 10^6^ human NSCs (LM-NS008) in 24 *μ*L of vehicle (VEH) (2% human serum albumin) or VEH alone on postoperative days 7, 9, 11, 13, 15, and 17. The administration procedure was as previously reported and shown to achieve distribution and efficacy of cellular therapy [23]. Subcutaneous injections of cyclosporin A (10 mg/kg; Alfa Aesar, Haverhill, MA) were administered daily to all rats beginning on postoperative day 5 for the duration of the study.

### 2.5. Cognitive Function: Acquisition of Spatial Learning and Memory Retention

Acquisition of spatial learning was assessed using a Morris water maze (MWM) task that has been shown to be sensitive to learning deficits after TBI [36–40]. Training was conducted every day on postoperative days 18–23. Briefly, the maze (180 cm diameter plastic pool) was filled with water (26 ± 1°C) to a depth of 28 cm and located in a room with visual cues saliently displayed on the walls. The escape platform was a clear Plexiglas stand (10 cm diameter) placed 2 cm below the surface of the water and positioned 26 cm from the edge of the pool in the southwest quadrant for all trials. Learning acquisition consisted of four trials, with random placement into the pool from one of each cardinal direction (north, east, south, and west). Each rat was given a maximum of 120 s on each trial to find the submerged platform. Rats that failed to find the platform in the allotted time were manually guided to and placed on the platform, where they remained for 30 s before being removed and placed in a warming chamber (ThermoCare®, Paso Robles, CA) for a 4 min intertrial interval. The mean of the 4 daily trials for each rat was used in the statistical analyses.

On postsurgery day 24, a single-probe trial was provided to evaluate memory retention. Briefly, during the probe trial, the platform was removed from the pool and the rat was placed in the maze at the location point most distal to the southwest quadrant where the platform was previously located (i.e., target quadrant) and allowed to freely explore the pool for 30 s. The percent time spent in the target quadrant was recorded and used in the statistical analysis. Lastly, after the probe trial, the platform was raised 2 cm above the water surface and a white tape was wrapped around the rim making it visible to the rats, which controls for visual acuity and nonspatial contributions like sensorimotor performance and motivation. The ANY-maze software tracking system (Stoelting, Wood Dale, IL) was used to automatically record all data, which included time to locate the platform, percent time in the target quadrant, and swim speed.

### 2.6. Generation and Characterization of Primary NSC Cultures

Isolation and propagation of fetal brain neural stem cells and immortalization with *L-MYC* gene have been previously described [41, 42]. All procedures that involved stem cell isolation and propagation were performed under approved City of Hope Institutional Review Board approved protocols (IRB #10079, SCRO #11002, and IBC #11016). LM-NSC008 cells were cultured in serum-free NSC medium (RHB-A medium; Stem Cell Science) supplemented with 10 ng/mL basic fibroblast growth factor (bFGF), 10 ng/mL epidermal growth factor (EGF), 2 mM L-glutamine (Invitrogen), and Gem21 NeuroPlex Serum-Free Supplement (GeminiBio-Products, #400-160). bFGF and EGF were added every other day, and the media were completely changed every 7 days. LM-NSC008 cells were modified using lentivirus to express enhanced green fluorescent protein (eGFP) and fLuc (LM-NSC008.GFP.fLuc) for visualization *in vivo* and in brain sections by fluorescent microscopy.

### 2.7. Expansion of LM-NSC008 Cells to Create Master Cell Banks

Generation and characterization of LM-NSC008 cells stably expressing *L-MYC* has been previously described [41]. Cells were passaged and characterized for up to passage 50 [42]. Briefly, LM-NSC008 cells were expanded to master cell banks using the Quantum Cell Expansion System (Terumo BCT) and frozen at a concentration of 2.4 × 10^7^ cells in CryoStore (BioLife Solutions) in liquid nitrogen. LM-NSC008 cells were thawed, washed with PBS, and administered via IN drops into rats bearing CCI or sham injury using previously optimized protocols [25, 43].

### 2.8. Brain Tissue Clearing (CLARITY) and Distribution of LM-NSC008 Cells

Rats received an overdose of sodium pentobarbital (i.p.) on day 25 (early timepoint) or day 46 (late timepoint) after surgery and then perfused with ice-cold 0.1 M phosphate-buffered saline and fixed with 4% PFA, and brains were harvested. Brains were prepared as thick (1 mm) sections using acrylamide-based tissue clearing (CLARITY) [44, 45]. Brain slices were then imaged by confocal microscopy (*z*-axis resolution) and examined for the presence of the LM-NSC008 cells by fluorescent microscopy.

For IHC, paraffin-embedded brain sections were sectioned into 10 *μ*m coronal sections and every 5th section was stained with anti-human Nestin antibody (Millipore; Cat# MAB5326, at 1 : 200 dilution) by the City of Hope Anatomic Pathology Core. Brain sections were then processed for antigen retrieval with Proteinase K (Dako ready-to-use Cat # S3020) and incubated in peroxidase quenching solution (0.3% H_2_O_2_ made in 100% methanol for 20 min at room temperature) and then in blocking solution (50% BlockAid, Invitrogen B10710; 50% Western Blocking Reagent, Roche Applied Sciences 11921673001; 1% Triton-100x). Sections were then stained with primary antibody in blocking solution and incubated overnight at 4°C, followed by several washes in PBS and reaction with biotinylated secondary antibody (1 : 250 dilution, Vector BA-2001) for 1 h as previously described [42]. Sections were washed and incubated in avidin-biotin complex (ABC) solution for 1 h at room temperature and 5 min in 3,3′-diaminobenzidine (DAB) substrate solution containing 0.25% H_2_O_2_. Finally, the brain sections were washed and mounted with Cytoseal 8 mounting media (Richard-Allan Scientific) and imaged.

### 2.9. Clarity Image Analysis

Maximum intensity projections of optically sectioned brain sections were generated using ImageJ. Background subtraction was performed with a 50-pixel radius. The resulting images were manually thresholded to generate the distribution of NSCs in the imaged tissue section. A mask of the tissue section was created using the maximum intensity projection of the entire brain. Segmented NSCs within the tissue mask were analyzed in MATLAB (MathWorks, MA). The location of TBI centroid was manually input, and the distance of each pixel identified as a part of an NSC from the TBI centroid was calculated. Additionally, based on the tissue mask, the distance of each of these pixels to the nearest edge of the tissue mask was calculated. The distribution of these pixels from the TBI site and the edge of the tissue is presented as histograms and cumulative distribution functions. Subsections of the images demonstrating the NSC presence were analyzed separately to evaluate the distance of NSCs from the TBI site (Supplemental Figs. S1, S2). For this purpose, instead of each pixel, clusters of pixels connected to each other were evaluated for distance to the TBI site.

### 2.10. Statistical Analyses

All behavioral analyses were performed using StatView 5.0.1 (Abacus Concepts Inc., Berkeley, CA) on data collected by blinded experimenters. Assessment of spatial learning was conducted with repeated measures analysis of variance (ANOVA). Probe trial, visible platform, and swim speed were analyzed using one-factor ANOVAs, as were the acute neurological outcomes (i.e., hind limb withdrawal reflex times and righting reflex times). When the overall ANOVA revealed significant effects, the Newman-Keuls post hoc test was used to determine specific group differences. The results are expressed as the mean ± standard error of the mean (S.E.M.) and are considered significant when *p* ≤ 0.05. Eight rats (males) died postsurgery, and thus, the statistical analyses were performed on the data from 40 rats that made up the final composition of groups: TBI + NSC (*n* = 15), TBI + VEH (*n* = 14), sham + NSC (*n* = 6), and sham + VEH (*n* = 5).

## 3. Results

### 3.1. Acute Neurological Function

No differences were observed between the groups randomized to TBI + NSC and TBI + VEH in the hind limb withdrawal reflex after a brief paw pinch or for return of the righting reflex following the cessation of anesthesia (*p* > 0.05; Table 1). The lack of significant differences with these acute neurological indices suggests that both TBI groups experienced an equivalent level of injury. Additionally, no differences were observed between the sham + NSC and sham + VEH rats on acute neurological outcomes (*p* > 0.05).

### 3.2. Spatial Distribution of in Administered LM-NSC008 Cells

**Figure 1:**
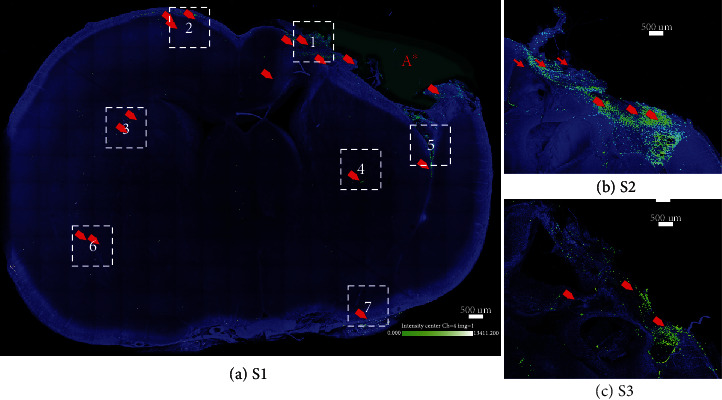
TBI-specific distribution of intranasally delivered LM-NSC008 cells in rats (day 25). (a) Confocal image (stitched at maximum intensity) of optically cleared coronal brain slice (1 mm thick). Rats that received either a TBI or sham injury and on days 7, 9, 11, 13, 15, and 17 post-surgery were IN-administered LM-NSC008 cells (1 × 10^6^ cells in 24 *μ*L). (a–c) On day 25, the rats were euthanized and 1 mm brain slices were cleared using CLARITY (PACT) and imaged using confocal microscopy. Three coronal sections (S1, S2, and S3) are shown overlapping the TBI site. LM-NSC008 cells were lentivirally transduced to express eGFP.Ffluc protein for visualization (LM-NSC008 cells—green and red arrows). LM-NSC008 cells highlighted and quantified within boxes 1–7 (the pseudo-object was drawn at TBI site A∗).

**Figure 2:**
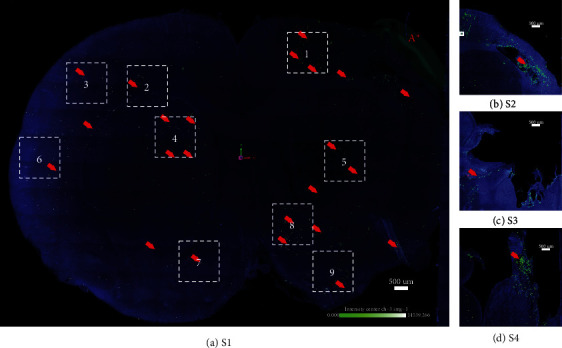
TBI-specific distribution of intranasally delivered LM-NSC008 cells in rats (day 46). (a) Confocal image (stitched at maximum intensity) of optically cleared coronal brain slice (1 mm thick). Rats that received either a TBI or sham injury and on days 7, 9, 11, 13, 15, and 17 post-surgery were IN-administered LM-NSC008 cells (1 × 10^6^ cells in 24 *μ*L). (a–d) On day 46, the rats were euthanized and 1 mm brain slices were cleared using CLARITY (PACT) and imaged using confocal microscopy. Coronal brain sections (S1, S2, S3, and S4) are shown overlapping the TBI site (LM-NSC008 cells—green and red arrows). LM-NSC008 cells highlighted in selected boxes 1–9 (the pseudo-object was drawn at TBI site A∗).

Within the brain parenchyma, imaging of LM-NSC008 cells was performed using confocal 3D imaging of optically cleared 1 mm thick brain sections at 25 and 46 days after TBI (early and late timepoints, respectively; Figures 1 and 2). Areas of brain tissue highlighted by the square boxes were used for the analysis of the number of cell clusters and the distance LM-NSC008 cells migrated from the TBI site. At the early timepoint, most LM-NSC008 cells were localized at the site of TBI damage in optically cleared sections (Figures 1(a)S1, 1(b)S2, and 1(c)S3), but at the later timepoint (day 46), LM-NSC008 cells were found at sites distant to the primary injury (Figures 2(a)S1, 2(b)S2, 2(c)S3, and 2(d)S4), indicating that LM-NSC008 cells migrate well beyond the lesion site, possibly following damaged axonal tracts.

**Figure 3:**
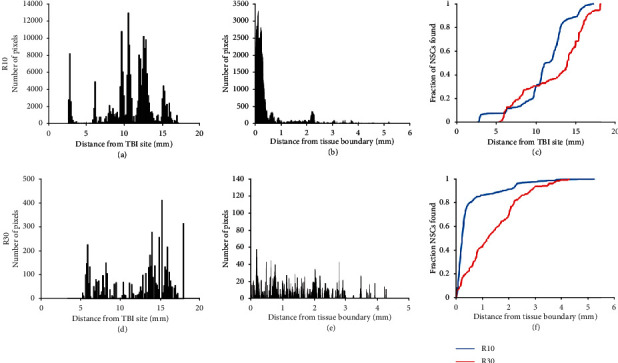
Quantification of distribution of LM-NSC008 cells at day 25 (R10) and day 46 (R30). (a, d) Distance LM-NSC008 cells migrated from the TBI site (A∗ in Figure 2 and this figure). (b, e) Distance quantified in combined sections (in mm) for LM-NSC008 cells migrated after intranasal administration for R10 (Figures 2(a)S1, 2(b)S2, and 2(c)S3) and R30 (S1, S2, S3, and S4). (c, f) Graphs represent migration dynamics of LM-NSC008 cells either from the TBI site or from the brain tissue boundary quantified at days 25 (blue curve) and 46 (orange curve). *y*-axis values are in millimeters (mm). Note there are an increased number of cells close to the TBI site and minimal migration at day 25 but there are fewer cells at the primary TBI site and greater migration at day 46. The data show that LM-NSC008 cells are migrating throughout the brain and into regions mediating the behaviors impacted by TBI, particularly during the later time which correlates with cognitive testing.

Total LM-NSC008 cell distributions in early and late timepoints are summarized from the data of R10 and R30, rats that were euthanized at days 25 and 46, respectively (Figure 3). Quantification of LM-NSC008 cell distributions was done by measuring distances from TBI sites and from the brain surface. The positions of the LM-NSC008 cells suggest that, conceivably, IN-administered NSCs could take neuronal (olfactory), vascular, or lymphatic migration routes to distribute in the subarachnoid space and then enter the brain parenchyma.

**Figure 4:**
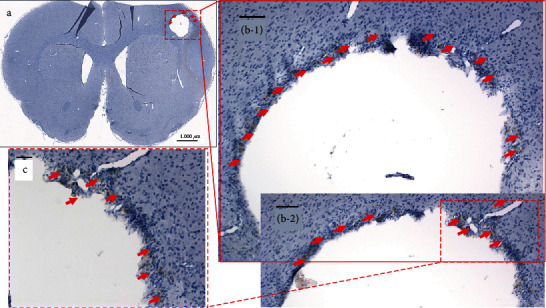
Immunohistochemistry staining using anti-human Nestin-specific antibodies to visualize LM-NSC008 cells at the TBI site on day 39 (female rats, coronal paraffin sections #64). (a) Bright field tile image of the rat brain with the CCI injury site —scale bar 1000 *μ*m. (b-1, b-2) Parallel coronal brain sections with LM-NSC008 cells stained with DAB brown and highlighted with red arrows, 10x. (c) 20x magnification LM-NSC008 cells at the CCI injury site. Scale bars 100 *μ*m and 50 *μ*m, respectively.

To probe for LM-NSC008 cells at the TBI site, female rat brains were harvested, fixed, sectioned, and stained for histological examination and IHC visualization of human Nestin on day 39 (Figure 4). LM-NSC008 (human Nestin-expressing) cells were found lining the edge of the CCI injury site (red arrows), at edges of the brain and within the brain parenchyma (Figures 4 and Fig. S3, red arrows).

### 3.3. Acquisition of Spatial Learning

**Figure 5:**
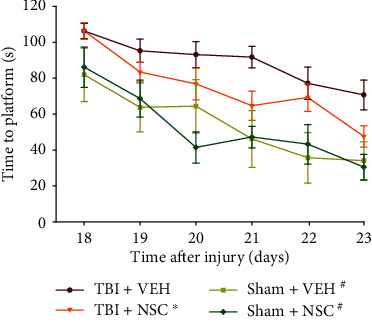
Beginning 1 week after TBI or sham injury, adult male rats were IN-administered LM-NSC008 cells (1 × 10^6^ in 24 *μ*L; TBI + NSC or sham + NSC) or VEH (2% human albumen serum; 24 *μ*L; TBI + VEH or sham + VEH) on post-surgery days 7, 9, 11, 13, 15, and 17 and then were evaluated for the acquisition of spatial learning in a well-established Morris water maze. Mean (±S.E.M.) time(s) to locate the escape platform over days 18–23. ^∗^*p* < 0.05 vs. TBI + VEH. ^#^*p* < 0.05 vs. TBI + VEH and TBI + NSC. Shams did not differ from one another *p* > 0.05.

Analysis of the spatial learning data revealed significant group (*F*_3,36_ = 9.37, *p* = 0.0001) and day (*F*_5,180_ = 18.07, *p* < 0.0001) differences. The TBI + NSC group learned the location of the submerged escape platform (i.e., acquired spatial learning) significantly better than the TBI + VEH group (*p* < 0.05). The time to locate the visible platform was also significantly longer for the TBI + VEH group relative to the sham + NSC and sham + VEH groups (*p* < 0.05; data not shown). Post hoc analysis revealed no differences between the sham + NSC and sham + VEH groups (*p* > 0.05), and both were better than the TBI + NSC and TBI + VEH groups (*p* < 0.05; Figure 5). Swim speed did not differ among the groups (range = 33.6 ± 1.7 cm/s to 34.8 ± 2.0 cm/s; *p* > 0.05). Also, no differences were observed among the groups in percent time spent in the target quadrant during the probe trial (*p* > 0.05; data not shown).

## 4. Discussion

These data indicate that IN administration of human LM-NSC008 cells after TBI significantly improves acquisition of spatial learning relative to VEH-treated TBI rats and that LM-NSC008 cells distribute throughout damaged brain tissue and into normal brain regions. That IN-administered LM-NSC008 cells were present in sufficient numbers to improve cognition is a significant finding as a major obstacle to the feasibility, and efficacy of NSC-based therapy is ensuring that enough numbers of viable cells reach the diseased or damaged areas in the CNS [23–25, 41]. Studies have shown that although IV-administered NSCs can cross the blood-brain barrier and localize to damaged tissue, they show limited accumulation in the brain (less than 1% of injected NSCs) [28]. IV administration of NSCs can also lead to systemic complications. IC administration of NSCs, while avoiding potential systemic reactions and resulting in engraftment of approximately 5–15% of injected cells [27], is invasive, costly, and potentially damaging to normal tissue [46].

Several groups have expended considerable effort designing and evaluating potential strategies to effectively restore damaged neuronal networks and recover cognitive function [47, 48]. However, developing these therapies has been hampered by limited access to relevant human tissue and the use of rodent models that imperfectly recapitulate human brain physiology [49]. Additionally, culture methods to create human stem cells have been developed, using embryonic stem cells (ESCs) and induced pluripotent stem cells (iPSCs) as starting materials, with iPSCs being the most common [49–51]. However, challenges have emerged when working with iPSCs, including variations in genetic backgrounds and phenotypes, which have limited their use as viable therapies [52–54]. In addition, iPSCs must be programmed to differentiate into specific cell types and this programming can be incomplete or erroneous, resulting in organoid heterogeneity, slow growth, and poor or incomplete differentiation [55–60]. However, the power of the use of allogeneic neural stem cells (LM-NSC008) has been demonstrated. LM-NSC008 cells can be further differentiated into region-specific neural cells and combined with other factors to produce complex brain structures including vascular, new synaptic-like junctions in 3D cultures and in vivo as this have been developed from human ESCs (hESCs) and iPSCs [61]. Other stem cell-based therapies investigated for the treatment of TBI or other neurodegenerative conditions include the use of NSCs derived from bone marrow-derived mesenchymal stem cells, dental pulp stem cells (DPSCs), embryonic stem cells, or umbilical cord-derived mesenchymal stem cells [62–75]. Indeed, DPSCs have been shown to be a favorable source of stem cells capable of differentiating into neuro-like cells and the possibility of using DPSCs in neurodegeneration was outlined in a several reports [76, 77]. Unlike LM-NSC008 cells that showed a cognitive benefit when administered alone after TBI, other NSC-based therapies produced a significant behavioral benefit only when combined with environmental enrichment (EE) in a rehabilitative strategy [69, 70, 78]. Based on previous combination therapies [72] and the efficacy of LM-NSC008 cells in the current study, combining LM-NSC008 cells with EE after TBI may work in synergy or additively to produce benefits even greater than that of LM-NSC008 cells alone.

Also, in rodent models, endogenous NSCs can generate new neurons and glial cells in regions of adult neurogenesis, such as the subventricular zone (SVZ) and dentate gyrus (DG) of the hippocampus [62, 65, 79]. Post-TBI stem cell recruitment to the SVZ and DG indicates inherent attempts of the brain to repair and regenerate after injury, which might be further mediated by administration of exogenous LM-NSC008 cells [66, 67]. Endogenous neurogenesis can be enhanced by introduction of exogenous growth factors, VEGF, statins, and progesterone, but stem cell therapies may more effectively enhance endogenous neurogenesis by integrating into host tissue as well as providing trophic support [68, 72, 80]. Gennai and others have demonstrated that stem cells and progenitor cells can migrate to the injured brain regions and proliferate, exerting protective effects through possible cell replacement and release of anti-inflammatory and growth factors in preclinical studies [81]. While stem/progenitor therapies demonstrated improvement after TBI and stroke in preclinical and clinical studies, exact mechanism(s) of the recovery is not well established. Some studies reported a limited migration ability in vivo; however, the benefits of cell-based therapy have been clearly demonstrated in the models of Parkinson's disease [46, 82]. Further investigation is required to determine whether brain repair occurs via cell replacement, immunomodulation, or endogenous tissue repair mechanisms [83].

By visualizing LN-NSC008 cells in situ, we have been able to correlate behavioral outcomes with the presence of NSCs at injury sites. Future studies should expand on these findings by evaluating strategies to enhance LM-NSC008 cell accumulation, including dosing paradigms. While not evaluated in this study, previous work from our laboratory has demonstrated that LM-NSC008 cells are not tumorigenic *in vivo* (tested for up to 12 months in immunodeficient mice), suggesting that they will be safe to use as a therapy for TBI [42]. Regarding possible sex differences, preliminary data from our laboratory shows that the benefits of LM-NSC008 cell therapy are equivalent in immunosuppressed female rats after TBI (unpublished).

These studies will have significant translational impact by providing a route of administration (intranasal) that is less invasive than other routes of stem cell administration and that will be more cost effective than current approaches because it can be done in an outpatient setting and can overcome risks and hindrances associated with current routes of administration. Thus, these findings have the potential to instill confidence in the use of NSC therapy that could ultimately serve as a viable treatment for the millions of TBI survivors who currently have few options for treatment of their debilitating motor and cognitive abilities.

Overall, the current study is the first to report an evaluation of distribution of allogeneic LM-NSC008 cells at the TBI sites and subsequent improvement in spatial learning. These studies, if expanded to other neurodegenerative diseases, could change the treatment paradigm for patients with TBI and other diseases of CNS, providing a method for noninvasive, repeat treatments.

## 5. Conclusions

Although previous studies have addressed IC and IN delivery of proteins, growth factors, hormones, small molecules, and stem cells to treat neurological disorders or glioma in rodents, those studies cannot be easily translated to humans without the use of expensive and time-consuming preclinical studies. To the best of our knowledge, the proposed studies will be the first to develop a quantitative model of human allogeneic NSC migration in the brain after IN delivery that can lead to optimization and prediction of the doses and routes of NSC delivery. These findings indicate that IN administration resulted in distribution of LM-NSC008 cells at TBI and distant to TBI sites in a rat model of CCI. LM-NSC008 cell distribution leads to improved recovery of cognitive performance, thereby demonstrating the potential utility of IN delivery of LM-NSC008 cells in a therapeutic context. This work also demonstrates the feasibility of using tissue clearing and volume imaging as a means of evaluating LM-NSC008 cell migration and distribution in the brain. These measures will aid in optimizing dose, timing, and route of NSC-mediated cellular therapies for clinical use.

## Figures and Tables

**Table 1:** Reflex behavior(s).

		TBI + LM − NSC008	TBI + VEH	
Withdrawal reflex	Left	167.0 ± 8.7	167.2 ± 4.3	*p* > 0.05
	Right	155.9 ± 4.9	162.6 ± 4.1	*p* > 0.05
Righting reflex		389.6 ± 19.2	395.9 ± 19.1	*p* > 0.05
		Sham + LM − NSC008	Sham + VEH	
Withdrawal reflex	Left	20.8 ± 3.1	18.0 ± .7	*p* > 0.05
	Right	13.2 ± 1.2	16.3 ± 2.9	*p* > 0.05
Righting reflex		127.7 ± 5.3	121.2 ± 6.4	*p* > 0.05

Mean ± S.E.M. acute neurological assessments. No significant differences were revealed between the TBI + LM − NSC008 and TBI + VEH groups in time(s) or between the sham + LM − NSC008 and sham + VEH groups to elicit a right and left hind paw reflexive withdrawal (after a brief paw pinch) or righting reflex after the cessation of anesthesia.

## Data Availability

The data are widely available for the research community.

## Supplementary Materials

Supplementary Materials Fig. S1: quantification of LM-NSC008 cell within boxes 1–7 at day 25. (A) Section is from day 25 post CCI (LM-NSC008s pseudocolored green, brain outlined blue). The TBI site is shown in the upper right quadrant (A∗ inset black and white image). (B–H) Number of cell clusters in boxes 1–7 (*y*-axis) versus distance of LM-NSC008 cells migrated from TBI site (*x*-axis). Fig. S2: (A–I) number of cell clusters quantified in each quadrant 1–9 (*y*-axis) vs. distance of LM-NSC008 cells migrated from the TBI site at day 46 post-TBI (*x*-axis). Fig. S3: immunohistochemistry staining using anti-human Nestin-specific antibodies to visualize LM-NSC008 cells at the TBI site on day 39 (female rats, coronal paraffin sections #70). (A) Bright field image of the CCI injury site, scale bar 1000 *μ*m. (B, C, D) 10x and 20x magnification of the lower left area of the CCI injury site. LM-NSC008 cells are brown and highlighted with red arrows. Scale bars 100 *μ*m and 50 *μ*m, respectively.
