# Supplementary material for: Intranasally Administered L-Myc-Immortalized Human Neural Stem Cells Migrate to Primary and Distal Sites of Damage after Cortical Impact and Enhance Spatial Learning
Source: Stem Cells Int. 2021 May 22;2021:5549381. doi: 10.1155/2021/5549381 (PMC8166475; doi:10.1155/2021/5549381)
Supplement: Supplementary Materials — Fig. S1: quantification of LM-NSC008 cell within boxes 1–7 at day 25. (A) Section is from day 25 post CCI (LM-NSC008s pseudocolored green, brain outlined blue). The TBI site is shown in the upper right quadrant (A∗ inset black and white image). (B–H) Number of cell clusters in boxes 1–7 (y-axis) versus distance of LM-NSC008 cells migrated from TBI site (x-axis). Fig. S2: (A–I) number of cell clusters quantified in each quadrant 1–9 (y-axis) vs. distance of LM-NSC008 cells migrated from the TBI site at day 46 post-TBI (x-axis). Fig. S3: immunohistochemistry staining using anti-human Nestin-specific antibodies to visualize LM-NSC008 cells at the TBI site on day 39 (female rats, coronal paraffin sections #70). (A) Bright field image of the CCI injury site, scale bar 1000 μm. (B, C, D) 10x and 20x magnification of the lower left area of the CCI injury site. LM-NSC008 cells are brown and highlighted with red arrows. Scale bars 100 μm and 50 μm, respectively. [file 5549381.f1.pptx]

## Slide 1
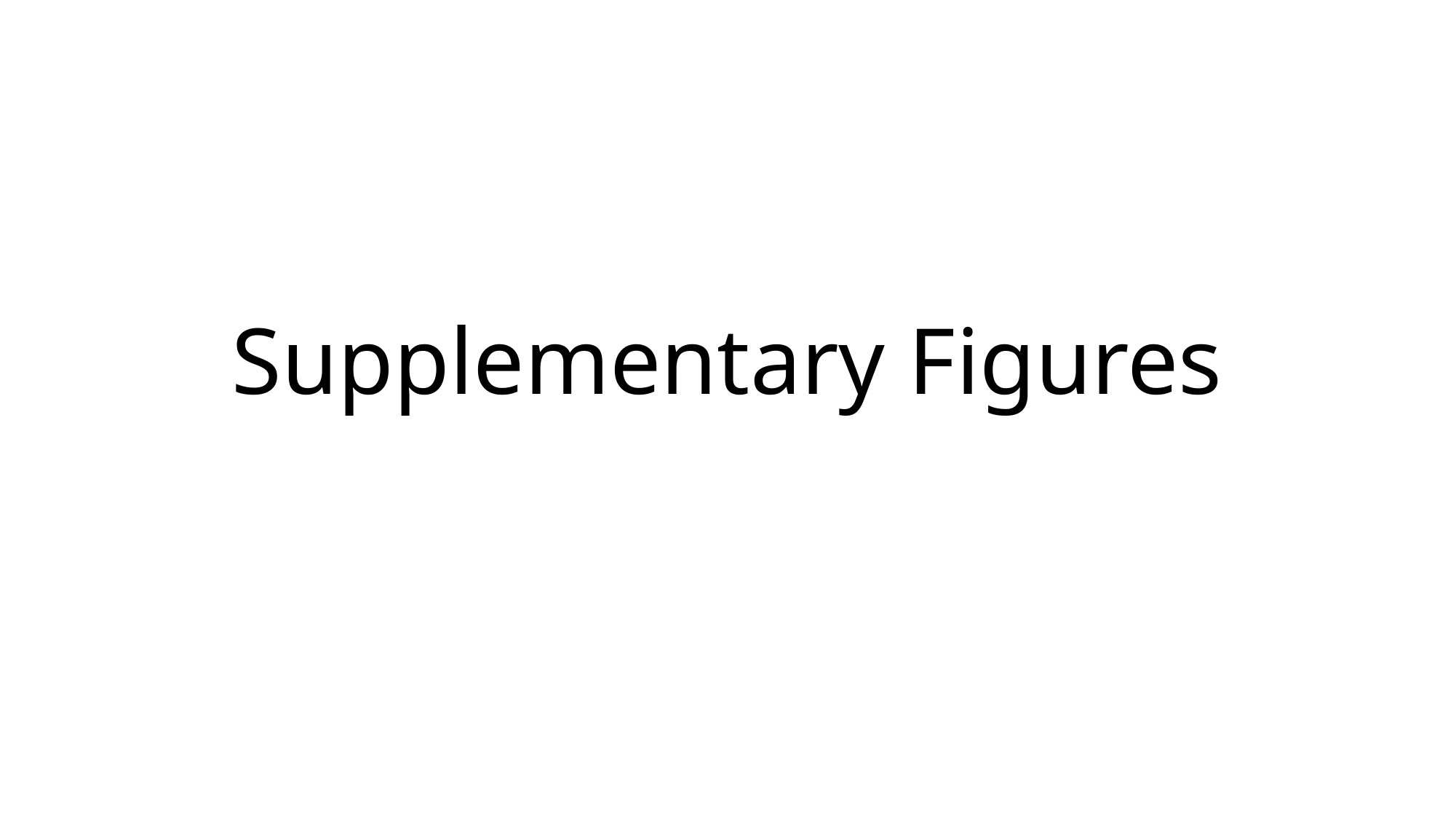

# Supplementary Figures

## Slide 2
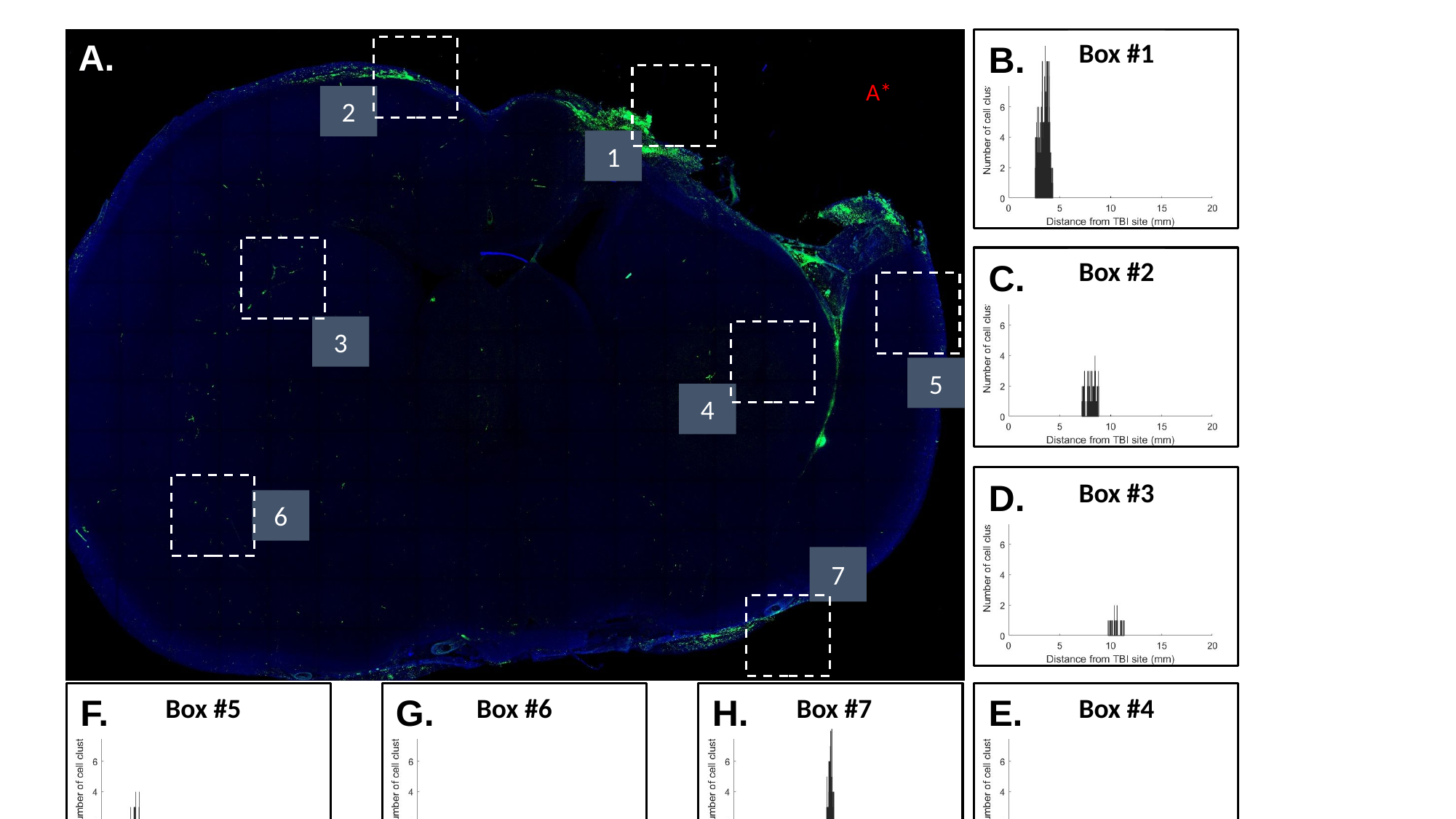

A.
A*
2
1
3
5
4
6
7
Box #1
B.
Box #2
C.
Box #3
D.
F.
Box #5
G.
Box #6
H.
Box #7
E.
Box #4

## Slide 3
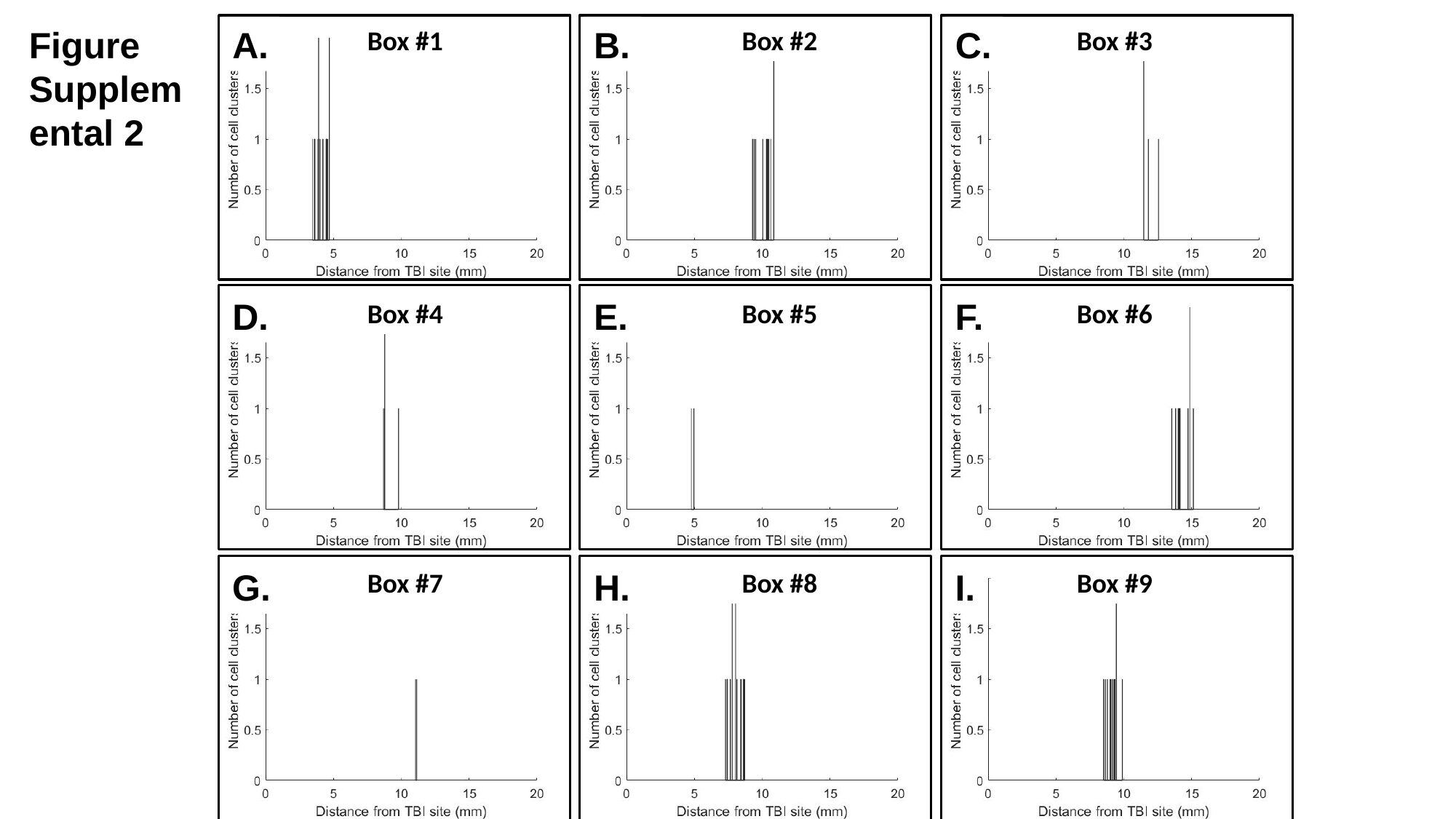

A.
B.
C.
Box #1
Box #2
Box #3
D.
E.
F.
Box #4
Box #5
Box #6
G.
H.
I.
Box #7
Box #8
Box #9
Figure Supplemental 2

## Slide 4
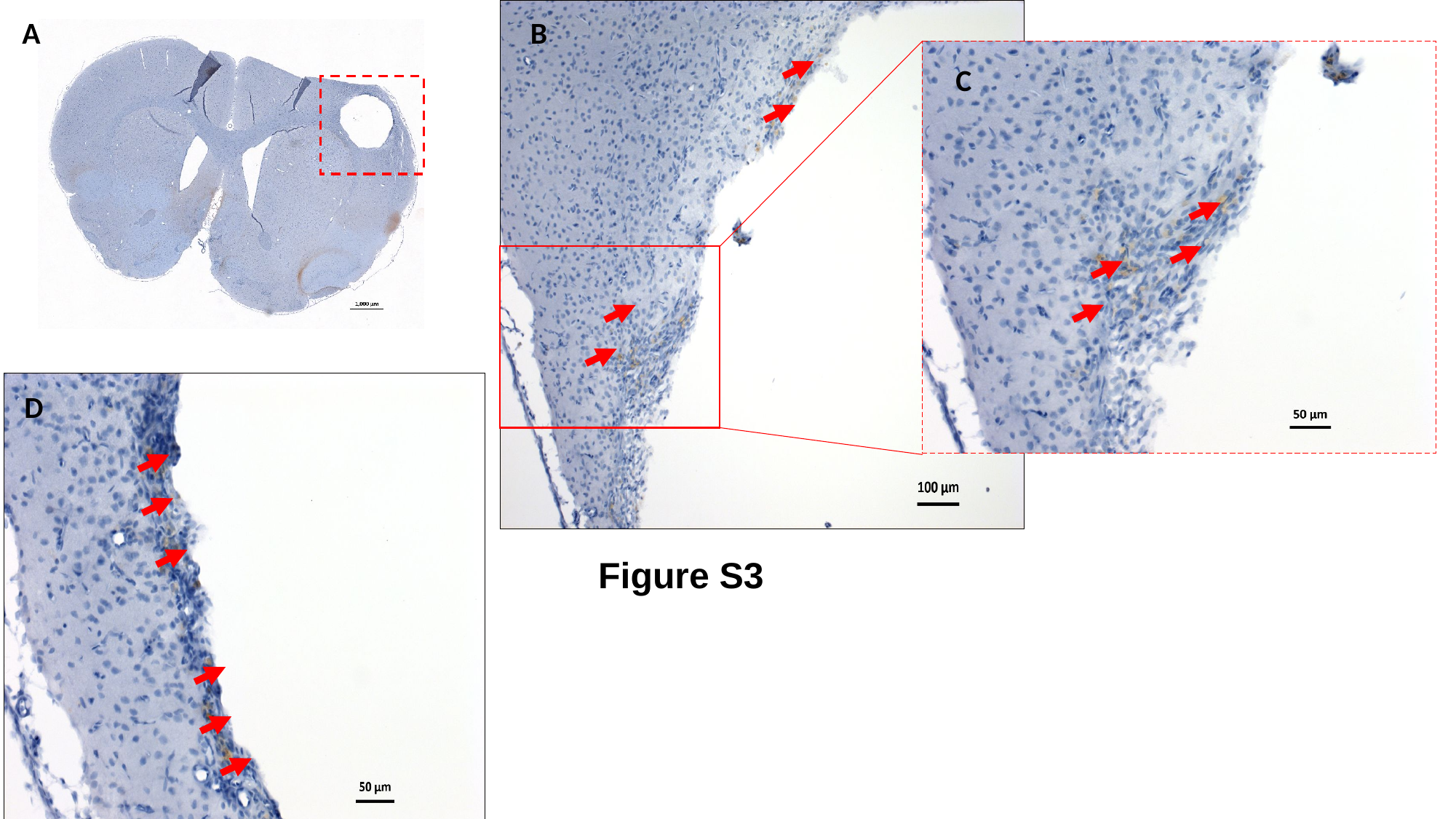

A
B
C
D
Figure S3
